# Supplementary material for: Identification of relevant genetic alterations in cancer using topological data analysis
Source: Nat Commun. 2020 Jul 30;11:3808. doi: 10.1038/s41467-020-17659-7 (PMC7393176; doi:10.1038/s41467-020-17659-7)
Supplement: Supplementary file 2 — Description of Additional Supplementary Files [file 41467_2020_17659_MOESM2_ESM.pdf]

Description of Additional Supplementary Files

File Name: Supplementary Data 1
Description: List of non-synonymous mutations for the significant genes in the integrative topological analysis of 12 tumor types.
