# Supplementary material for: Identification of relevant genetic alterations in cancer using topological data analysis
Source: Nat Commun. 2020 Jul 30;11:3808. doi: 10.1038/s41467-020-17659-7 (PMC7393176; doi:10.1038/s41467-020-17659-7)
Supplement: Supplementary file 4 — Reporting Summary [file 41467_2020_17659_MOESM4_ESM.pdf]

nature research

Corresponding author(s): Pablo G. Camara, Arnold J. Levine, Raul Rabadan

Last updated by author(s): Jun 26, 2020

# Reporting Summary

Nature Research wishes to improve the reproducibility of the work that we publish. This form provides structure for consistency and transparency in reporting. For further information on Nature Research policies, see our Editorial Policies and the Editorial Policy Checklist.

## Statistics

For all statistical analyses, confirm that the following items are present in the figure legend, table legend, main text, or Methods section.

| n/a | Confirmed | |
|---|---|---|
| ☐ | ☒ | The exact sample size (*n*) for each experimental group/condition, given as a discrete number and unit of measurement |
| ☐ | ☒ | A statement on whether measurements were taken from distinct samples or whether the same sample was measured repeatedly |
| ☐ | ☒ | The statistical test(s) used AND whether they are one- or two-sided<br>*Only common tests should be described solely by name; describe more complex techniques in the Methods section.* |
| ☒ | ☐ | A description of all covariates tested |
| ☐ | ☒ | A description of any assumptions or corrections, such as tests of normality and adjustment for multiple comparisons |
| ☐ | ☒ | A full description of the statistical parameters including central tendency (e.g. means) or other basic estimates (e.g. regression coefficient) AND variation (e.g. standard deviation) or associated estimates of uncertainty (e.g. confidence intervals) |
| ☐ | ☒ | For null hypothesis testing, the test statistic (e.g. *F*, *t*, *r*) with confidence intervals, effect sizes, degrees of freedom and *P* value noted<br>*Give P values as exact values whenever suitable.* |
| ☒ | ☐ | For Bayesian analysis, information on the choice of priors and Markov chain Monte Carlo settings |
| ☒ | ☐ | For hierarchical and complex designs, identification of the appropriate level for tests and full reporting of outcomes |
| ☐ | ☒ | Estimates of effect sizes (e.g. Cohen's *d*, Pearson's *r*), indicating how they were calculated |

*Our web collection on statistics for biologists contains articles on many of the points above.*

## Software and code

Policy information about availability of computer code

| | |
|---|---|
| Data collection | No software was used for data collection. |
| Data analysis | Custom code has been deposited online (https://github.com/CamaraLab/TDA-TCGA/). Ayasdi v7.0 (https://www.ayasdi.com/platform/) was used to build topological representations. |

For manuscripts utilizing custom algorithms or software that are central to the research but not yet described in published literature, software must be made available to editors and reviewers. We strongly encourage code deposition in a community repository (e.g. GitHub). See the Nature Research guidelines for submitting code & software for further information.

## Data

Policy information about availability of data

All manuscripts must include a data availability statement. This statement should provide the following information, where applicable:

- Accession codes, unique identifiers, or web links for publicly available datasets
- A list of figures that have associated raw data
- A description of any restrictions on data availability

The gene expression data that support the results of this study are available at the TCGA repository (https://portal.gdc.cancer.gov/). The complete list of RSEM files used from this portal can be found in Supplementary Table 4. The somatic mutation data and MutSig2CV results that support the results of this study are available at the Broad Institute TCGA GDAC Firehose Portal (http://gdac.broadinstitute.org/). The complete list of MAF and MutSig2CV files used from this portal can be found in Supplementary Table 4. Representative topological representations have been deposited in an online database for each of the 12 tumor types (https://rabadan.c2b2.columbia.edu/pancancer). Raw data supporting the results presented in figure 3 are provided as a Source Data file.

# Field-specific reporting

Please select the one below that is the best fit for your research. If you are not sure, read the appropriate sections before making your selection.

☒ Life sciences ☐ Behavioural & social sciences ☐ Ecological, evolutionary & environmental sciences

For a reference copy of the document with all sections, see nature.com/documents/nr-reporting-summary-flat.pdf

# Life sciences study design

All studies must disclose on these points even when the disclosure is negative.

| | |
|---|---|
| Sample size | Sample sizes were determined by TCGA data availability. We used all the patients for which both gene expression and somatic mutation data were available. We estimated the power of our approach by randomly sub-sampling patients and computing the number of significant mutations at a fixed false discovery rate, as described in the manuscript. |
| Data exclusions | No data was excluded from the analyses. |
| Replication | Cell proliferation assays were performed in 6 replicates. Invasion assays were performed in 3 independent experiments with 3 replicates for each condition. Mouse model experiments were performed in 17 mice for each condition. |
| Randomization | Female and male mice were allocated on equal proportions across conditions. We performed a Mann-Whitney U-test to test for differences in the number of tumors between mice of different sex in each condition, we did not find any significant difference (p-values > 0.8 in all cases). |
| Blinding | Investigators were blinded to group allocation during data collection. |

# Reporting for specific materials, systems and methods

We require information from authors about some types of materials, experimental systems and methods used in many studies. Here, indicate whether each material, system or method listed is relevant to your study. If you are not sure if a list item applies to your research, read the appropriate section before selecting a response.

## Materials & experimental systems

| n/a | Involved in the study | |
|---|---|---|
| ☐ | ☒ | Antibodies |
| ☐ | ☒ | Eukaryotic cell lines |
| ☒ | ☐ | Palaeontology and archaeology |
| ☐ | ☒ | Animals and other organisms |
| ☒ | ☐ | Human research participants |
| ☒ | ☐ | Clinical data |
| ☒ | ☐ | Dual use research of concern |

## Methods

| n/a | Involved in the study | |
|---|---|---|
| ☒ | ☐ | ChIP-seq |
| ☒ | ☐ | Flow cytometry |
| ☒ | ☐ | MRI-based neuroimaging |

## Antibodies

| | |
|---|---|
| Antibodies used | Anti-ADAMTS12 (Santa Cruz Biotechnologies, H-142, sc-25583), anti-Ki67 (Abcam, ab66155), anti-ACTB (Sigma-Aldrich, AC-15, A1978), HRP-peroxidase-labelled anti-rabbit (Cell Signalling Technology, 7074S), HRP-peroxidase-labelled anti-mouse (Jackson ImmunoResearch, 115-035-062) |
| Validation | Antibodies have been validated by the manufacturer and used for the same species and application in multiple publications. Anti-ACTB: Gimona, M., et al., Cell Motil. Cytoskel., 27, 108-116 (1994) and North, A.J., et al., J. Cell Sci., 107, 445-455 (1994). Anti-ADAMTS12: Zhang, Q., Ji, Q., et al., Osteoarthritis and Cartilage/OARS 23, 2259-2268 (2015). Anti-Ki67: Ksander B et al., Nature. 511, 353-357 (2014). |

## Eukaryotic cell lines

Policy information about cell lines

| | |
|---|---|
| Cell line source(s) | LL/2-luc-M38 (Caliper) |
| Authentication | We did not authenticate the LL/2-luc-M38 cell line as it is a commercial cell line. |
| Mycoplasma contamination | Cell line was commercially acquired from manufacturer (Caliper). Authentication and contamination controls were performed by manufacturer as described in their website. |

| | |
|---|---|
| Commonly misidentified lines (See ICLAC register) | No commonly misidentified cell lines were used in this study. |

## Animals and other organisms

Policy information about studies involving animals; ARRIVE guidelines recommended for reporting animal research

| | |
|---|---|
| Laboratory animals | Wild-type mus musculus (C57BL/6J, male and female, age 6-8 weeks). ADAMTS12-/- mus musculus (C57BL/6J, male and female, age 6-8 weeks), generated in El Hour et al. Oncogene 29, 3025-32 (2010). |
| Wild animals | This study did not involve wild animals. |
| Field-collected samples | This study did not involve animals collected from the field. |
| Ethics oversight | Mouse experiments were performed following the institutional guidelines of the University of Oviedo (Comité de Ética en Experimentación Animal). |

Note that full information on the approval of the study protocol must also be provided in the manuscript.
